# Natural History of Invasive Papillary Breast Carcinoma Followed for 10 Years: A Case Report and Literature Review

**DOI:** 10.1155/2017/3725391

**Published:** 2017-06-08

**Authors:** Yong Joon Suh, Hyukjai Shin, Tae Jung Kwon

**Affiliations:** ^1^Department of Breast and Endocrine Surgery, Hallym University Sacred Heart Hospital, Anyang, Republic of Korea; ^2^Department of Surgery, Myongji Hospital and Seonam University College of Medicine, Goyang, Republic of Korea; ^3^Department of Pathology, Myongji Hospital and Seonam University College of Medicine, Goyang, Republic of Korea

## Abstract

Diachronic research on untreated breast cancer completely depends on past medical records when no more recent, advanced methods are available. Herein, we report a case of invasive papillary breast carcinoma followed for 10 years in a 59-year-old woman who refused any treatment. The diagnosis was based on core needle biopsies. At the patient's first visit in July 2006, the tumor measured 10.4 × 7.2 × 3.5 cm. It was staged as IIIB (T4bN1). In May 2016, the tumor was staged as IIIC (T4bN3a). In the past 10 years, the tumor has increased to 12.1 × 9.0 × 4.2 cm. However, a whole-body bone scan and ^18^F-FDG PET/CT showed no evidence of distant metastasis. Immunohistochemistry results, corresponding to biopsies taken at subsequent examinations, have remained unaltered since 2006. The tumor was estrogen/progesterone receptor-positive and C-erbB2 expression was not detected. The Ki-67 labeling index was around 10%.

## 1. Introduction

A multidisciplinary approach to breast cancer has led to a decrease in the number of patients who refuse treatment [1]. These days, few patients with breast cancer refuse therapy. Studies on the natural history of untreated breast cancer completely depend on past medical records when no more recent, advanced methods are available [1, 2]. It is difficult to obtain long-term follow-up results, especially for rare histological subtypes of breast cancer, such as invasive papillary carcinoma. However, an understanding of disease progression enables clinicians to alter the clinical course of breast cancer and prevent the development of more serious consequences. Herein, in addition to a literature review, we report a case of invasive papillary breast carcinoma in a patient who refused treatment along with data from 10 years of follow-up. The patient provided informed consent for the publication of this report.

## 2. Case Presentation

**Figure 1:**
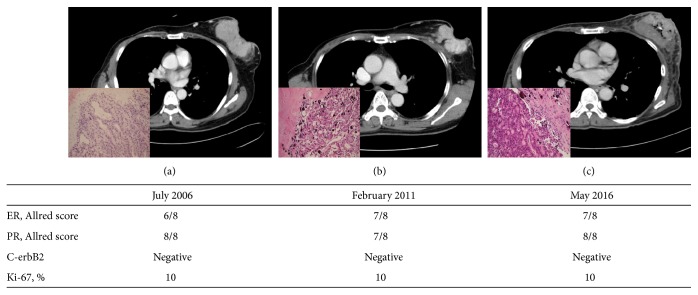
(a–c) The progression of invasive papillary breast carcinoma followed up for 10 years (LM, ×200). ER: estrogen receptor; PR: progesterone receptor.

**Figure 2:**
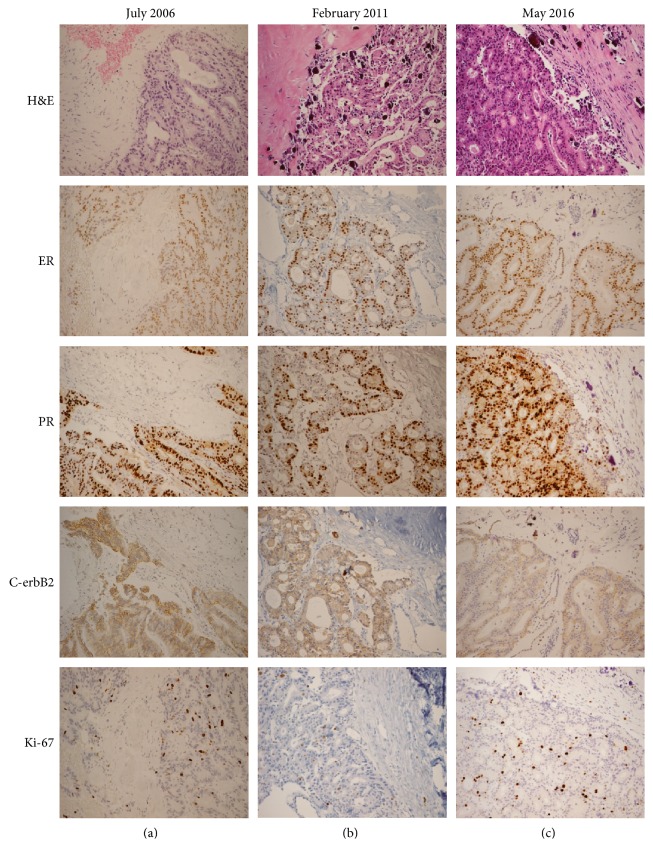
(a–c) Unaltered immunohistochemistry (ER/PR/C-erbB2/Ki-67) as an independent prognostic factor (LM, ×200). H&E: hematoxylin and eosin; ER: estrogen receptor; PR: progesterone receptor.

A 59-year-old woman visited our hospital in May of 2016 complaining of a palpable mass in the left breast. The postmenopausal patient denied any underlying disease, hormonal medication, or family history of breast cancer. A huge mass was noted on the upper outer area of the breast tissue. Skin abutting the tumor showed progression to necrosis with a foul-smelling discharge. Light microscopy revealed that the tumor had a predominantly papillary architecture with the papillae formed by malignant epithelial cells intimately related to fine fibrovascular cores. The patient had been diagnosed with invasive papillary breast carcinoma (G2), based on radionuclear and pathologic assessments. In 1996, the patient underwent right mastectomy in another hospital, but detailed records were not available. However, this time, despite constant counseling by medical personnel, the patient refused to receive any treatment except for follow-up examinations. The patient feared recurrence or metastasis due to treatment. At the patient's first visit to our hospital in July 2006, the tumor measured 10.4 × 7.2 × 3.5 cm and was accompanied by punctate hemorrhage. It was staged as IIIB (T4bN1) according to the 7th edition of the AJCC staging system because of two enlarged lymph nodes (diameter range, 1.3–1.5 cm) in the axillary vein group. The patient had received only conservative or supportive care. In the past 10 years, the size of tumor increased to 12.1 × 9.0 × 4.2 cm and included multifocal air-bubble portions (Figure 1). Over time, more prominent lymph nodes (diameter range, 1.5–1.9 cm) were observed in the subclavicular group, as well as along the axillary lymphatic chain. The tumor was staged as IIIC (T4bN3a) in 2016. However, a whole-body bone scan and ^18^F-FDG PET/CT showed no evidence of distant metastasis. The stained slides were independently examined by a reference pathologist. The immunohistochemistry results, corresponding to biopsies taken at subsequent examinations, have remained unaltered since 2006 (Figure 2). The tumor was estrogen/progesterone receptor-positive and C-erbB2 expression was not detected. The Ki-67 labeling index was around 10%.

## 3. Discussion

Papillary breast carcinoma is a rare type of breast cancer, accounting for less than 1% of all breast cancer cases [3]. It has a favorable prognosis, which was evident in the present case [4]. Lymph node involvement and distant metastasis are uncommon [5]. It is predominantly seen in postmenopausal women [6]. Histological characterization reveals proliferation of cells arranged around fibrovascular cores, grossly forming a circumscribed mass [7]. It is important to differentiate invasive papillary carcinoma from noninvasive forms. Moreover, invasive nonpapillary carcinoma associated with encapsulated papillary carcinoma and solid papillary carcinoma should not be classified as invasive papillary carcinoma but instead categorized according to the individual invasive component [5].

All malignant papillary proliferation cases of the breast lack an intact myoepithelial cell layer within the papillae. This important feature allows distinction from cases of benign papilloma [8]. In assessing the presence of a complete myoepithelial layer, p63 is often used as an adjunct to assess the presence and distribution of myoepithelial cells in papillary neoplasms of the breast [9]. Other immunohistochemical markers, such as estrogen/progesterone receptor, C-erbB2, and Ki-67, provide prognostic information [10]. Papillary breast carcinoma is usually estrogen/progesterone-receptor-positive and C-erbB2-negative, as demonstrated by immunohistochemical results in the present case [11]. These molecular expressions correspond with the luminal A-like subtype, which is associated with a lower recurrence rate and longer disease-free interval [10]. Interestingly, in the present case, the patient's immunohistochemical analysis remained unaltered for 10 years. Alterations in biomarker expression during disease progression have been reported in relatively few studies; these include studies on the effect of neoadjuvant chemotherapy on biomarker expression and the differences in biomarker expression between the primary cancer and corresponding metastases [12, 13]. By analyzing paired primary cancer and corresponding asynchronous metastases during the metastatic process of breast cancer, Kümler et al. [12] reported the discordant expression in biomarkers such as estrogen, C-erbB2, Ki-67, p53, Bcl-2, TOP2a, and TOP1. Similarly, one meta-analysis found that estrogen/progesterone receptor status in breast cancer was altered significantly even by neoadjuvant chemotherapy [13]. No firm conclusions have yet been drawn. The relationship between disease prognosis and immunohistochemical alterations in breast cancer needs to be investigated in future trials.

In terms of natural history, breast cancer is a progressive disease which should be prevented by screening [2]. With an increase in awareness of breast cancer, studies on the natural history of untreated breast cancer necessarily depend on past medical records (Table 1). In one pioneering study, 250 patients with untreated breast cancer were enrolled between 1805 and 1933 [1]. The vast majority of cases were advanced (stage III/IV: 97.6%). From the time of symptom onset, the mean duration of survival was 3 years and the median duration of survival was 2.7 years. Additionally, the study included long-term survivors. The longest survivor lived for 18.3 years. However, the factors associated with prolonged survival could not be explained even in modern times because no radionuclear examinations or adjunctive immunohistochemical techniques were available. The present case compensates for the shortcomings of the previous study.

In the present case, the patient experienced asynchronous contralateral breast cancer after mastectomy. Contralateral breast cancers occur at a rate of 0.5% to 1% yearly [14]. The risk of developing a second primary breast cancer in the contralateral breast is 3–5 times higher than that of developing a first primary breast cancer [15, 16]. In patients with primary stage I/II breast cancer, the average annual hazard rate for the contralateral breast cancer was 0.8% in a follow-up study done over 20 years [17]. In the Netherlands, the cumulative incidence increased by 0.4% per year, reaching 5.9% after 15 years [18]. The mortality rate of patients diagnosed with asynchronous contralateral breast cancer was found to be 44% (95% confidence interval, 33–56%) higher than the mortality rate of patients without asynchronous contralateral breast cancer in the same study. Therefore, patients who have received treatment for primary breast cancer require careful clinical examination supplemented by mammography.

The present report has a few limitations. It depended on core needle biopsies, although a specific, detailed diagnosis should be made after complete surgical resection. In addition, more diverse ancillary techniques were not tested.

## 4. Conclusion

The standard treatment in invasive papillary breast carcinoma is surgery as lumpectomy, although invasive papillary breast carcinoma shows a relatively slow disease progression as noted in the present case. It remains a high risk to observe only. The current case is also a reminder that patients who have received treatment for primary breast cancer require careful clinical examination supplemented by mammography.

## Figures and Tables

**Table 1:** Literature review on the natural history of untreated breast cancer. Adapted from H. J. Bloom, W. W. Richardson, and E. J. Harries, “Natural history of untreated breast cancer (1805–1933). Comparison of untreated and treated cases according to histological grade of malignancy.” Br Med J, vol. 2, no. 5299, p. 213, 1962 [1].

Authors	Year	Number	Area	Period	Mean duration of life^*∗*^	Median duration of life^*∗*^
Bloom	1962	250	UK	1805–1933	35.5	32.4
Wade	1946	26	UK	1931–1941	32.6	—
Nathanson	1936	100	USA	1912–1932	—	30
Forber	1931	64	UK	1928-1929	39.3	—
Daland	1927	100	USA	—	40.5	30
Greenwood	1926	651	UK	—	38.4	27.6

^*∗*^Months.
